# Laminaran Attenuates NaCl-Induced Cytotoxicity via ROS Scavenging and Prevents Alteration of Cellular Elastic Modulus

**DOI:** 10.3390/md24050179

**Published:** 2026-05-15

**Authors:** Hiromi Kurokawa, Atsushi Taninaka, Hirofumi Matsui, Hidemi Shigekawa, Yutaka Kuroki, Makoto M. Watanabe

**Affiliations:** 1 PhycoChemy Corporation, 4-19-1, Midorigahara, Tsukuba 300-2646, Ibaraki, Japan; m-watanabe@phycochemy.jp; 2 Algae Biomass Energy System R&D Center, University of Tsukuba, 1-1-1 Tennodai, Tsukuba 305-8575, Ibaraki, Japan; hmatsui@md.tsukuba.ac.jp; 3 Faculty of Medicine, University of Tsukuba, 1-1-1 Tennodai, Tsukuba 305-8575, Ibaraki, Japan; 4 Institute of Pure and Applied Sciences, University of Tsukuba, 1-1-1 Tennodai, Tsukuba 305-8573, Ibaraki, Japan; jun_t@bk.tsukuba.ac.jp (A.T.); hidemi@ims.tsukuba.ac.jp (H.S.); 5 Takano Co., Ltd., Miyada-mura, Kamiina-gun, Nagano 399-4301, Nagano, Japan; 6 Delightex Pte. Ltd., 230 Victoria Street, #15-01 Bugis, Junction Towers, Singapore 188024, Singapore; yutaka.kuroki@jt.com

**Keywords:** laminaran, NaCl, reactive oxygen species, antioxidant, atomic force microscopy

## Abstract

Salt is essential for the maintenance of cellular homeostasis and transmission of nerve impulses. However, excessive salt intake (especially NaCl) causes hypertension and neoplasms and is associated with neoplasms, including esophageal and gastric cancer. High concentrations of NaCl enhances intracellular reactive oxygen species (ROS) production, especially that of superoxide anions (O_2_^−^), and induces injury to rat gastric mucosal cells (RGM1). In contrast, cells overexpressing manganese superoxide dismutase exhibit attenuated NaCl-induced cytotoxicity. Therefore, antioxidants can reduce the risk of salt-induced gastric mucosal injury. NaCl also affects the remodeling of the cytoskeleton and lamellipodia, and potentially modulates the cellular elastic modulus. In this study, we aimed to determine the possibility of cellular physiological changes by NaCl treatment and the effect of antioxidant laminaran in attenuating NaCl-derived cytotoxicity. Our in vitro assay revealed that laminaran attenuated NaCl-induced cytotoxicity and reduced intracellular ROS production caused by NaCl exposure. Laminaran upregulated antioxidant enzyme expression, suggesting that the observed reduction in ROS was mediated, at least in part, by the activation of these enzymes. Moreover, apoptosis derived from NaCl was inhibited by laminaran. NaCl also induced changes in lamellipodia formation; however, laminaran suppressed this formation.

## 1. Introduction

Salt is necessary for the maintenance of cellular homeostasis and transmission of nerve impulses. However, excessive salt consumption (especially NaCl) causes several diseases and is a major global problem [1]. High salt intake induces hypertension by in creasing blood volume, decreasing kidney function, and impairing renin–angiotensin–aldosterone-system reaction [2]. The effects of salt on the gastrointestinal tract, especially the stomach, increase the risk of gastric cancer [3]. The stomach is protected by gastric juice composed of hydrochloric acid, pepsinogen, and mucus; however, salt stimulation destroys the gastric wall barrier created by the gastric juice. This disruption can lead to gastritis, which in turn increases the risk of developing gastric cancer [2]. Additionally, damage to gastric mucosa increases the risk of bacterial infections, such as *Helicobacter pylori*, which synergistically increases the risk of gastric cancer [4]. Gastric cancer is a serious disease worldwide, with 784,000 deaths reported in 2018 [5]. We previously investigated NaCl-derived cytotoxicity with a focus on reactive oxygen species (ROS) and found that high concentrations of NaCl can enhance intracellular ROS production, especially that of superoxide anions (O_2_^−^), and induced cell injury in normal rat gastric mucosal cells [6]. These results imply that NaCl induces necrosis factors in gastric mucosal epithelial cells and other cell death signals due to oxidative stress. Intracellular ROS production and NaCl-derived cytotoxicity were attenuated in cells overexpressing O_2_^−^ scavenging enzymes [6]. These results indicate that combining salt intake with antioxidants may reduce the risk of gastric mucosal injury.

Moreover, NaCl affects the remodeling of the cytoskeleton and lamellipodia [7]. Cytoskeletal remodeling is regulated by direct interactions with members of the Rho family of small GTPases such as Rac1 and RhoA [8]. The Rac1 pathway contributes to lamellipodia formation via the WAVE and Arp2/3 complexes. Lamellipodia contains a dense network of actin filaments and drive cell migration [9]. Stress fibers are composed of actin filaments and myosin, which contribute to cytoskeleton organization and the maintenance of tensional homeostasis via the RhoA pathway [8,9,10]. The cellular elastic modulus of the fiber structure is increased compared to areas without fibers. It is decreased by reagents inhibiting actin polymerization [11]. Lamellipodia formation is dependent on actin filaments [12]; therefore, the elastic modulus of lamellipodia induced by NaCl may increase.

Seaweed contains many nutrients such as minerals, vitamins, and soluble dietary fiber, which reduce the risk of lifestyle-related diseases [13]. Laminaran is a water-soluble polysaccharide found in seaweeds and is composed of β-1,3/1,6-glucans [14]. Sources of β-1,3/1,6-glucans include yeasts, mushrooms, fungi, and seaweeds [15]. In particular, β-1,3/1,6-glucans derived from black yeast *Aureobasidium pullulans* exhibit several beneficial effects such as intestinal immunity, inflammation reduction, intestinal protection, and antitumor, anti-virus, and anti-allergy activities [16]. The functional activities of laminaran include antitumor, anti-inflammatory, and immunostimulatory activity [17]; however, the number of studies on laminaran is less than that of those on black yeast. We reported that laminaran scavenges ROS and reduces cytotoxicity induced by drug administration [18]. We hypothesized that the antioxidant effects of laminaran suppress salt-related cytotoxicity by suppressing ROS production.

In this study, using in vitro assays, we demonstrated two key findings: (1) the ability of laminaran to attenuate NaCl-induced cytotoxicity by scavenging ROS, and (2) the impact of NaCl treatment on cellular physiological alterations.

## 2. Results

### 2.1. Laminaran Attenuates Cytotoxicity of NaCl

**Figure 1:**
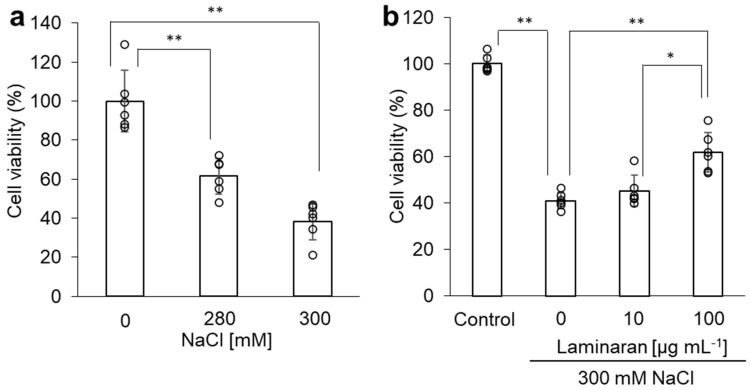
The cytotoxicity effect of NaCl. The cells were incubated in a medium containing NaCl with or without laminaran for 24 h. (**a**) NaCl showed cytotoxicity in a dose-dependent manner. (**b**) Laminaran suppressed the cytotoxicity derived NaCl. Data are expressed as means ± SD (*n* = 6). * *p* < 0.05, ** *p* < 0.01.

The cytotoxicity of NaCl in RGM1 cells was evaluated by the WST assay. Cell viability of 280 mM or 300 mM NaCl treated cells was significantly decreased from 61.4 ± 9.3 to 38.5 ± 9.5%, compared to NaCl-untreated cells, in a dose-dependent manner (Figure 1a). When cells were incubated with 10 µg/mL or 100 µg/mL laminaran in the presence of 300 mM NaCl, the cell viability of 100 µg/mL laminaran-treated cells was 61.9 ± 8.6%, which was significantly higher than that of laminaran-untreated cells (41.0 ± 3.5%) (Figure 1b). These results indicate that NaCl-induced cytotoxicity increased in a dose-dependent manner, although suppressed by the action of laminaran.

### 2.2. Laminaran Scavenges Intracellular ROS Derived from NaCl

**Figure 2:**
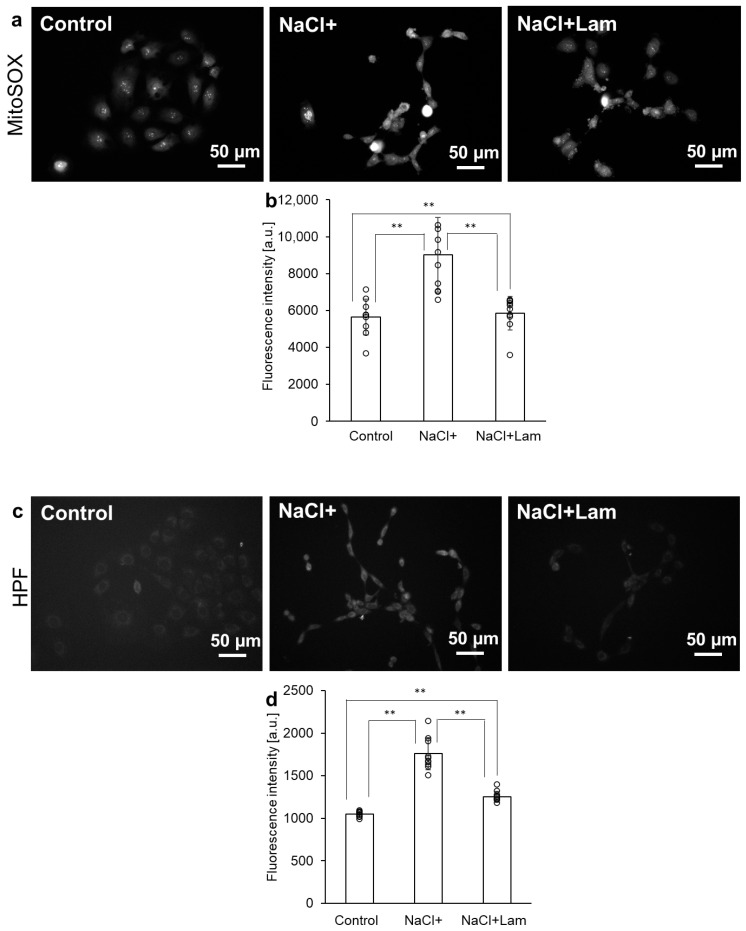
Intracellular ROS production derived from NaCl. The fluorescence intensity of MitoSOX and HPF were measured using fluorescence microscopy. Fluorescence intensity of MitoSOX is shown in (**a**,**b**) and that of HPF is shown in (**c**,**d**). Data are expressed as means ± SD (*n* = 10). ** *p* < 0.01.

MitoSOX and HPF fluorescent dyes were used to evaluate NaCl-induced intracellular ROS production. MitoSOX detects O_2_^−^, while HPF detects hydroxyl radical (OH^−^) and peroxynitrite (ONOO^−^). Use of these fluorescent dyes enabled the estimation of the ROS levels induced by NaCl. Cells were incubated for 1 h in a medium containing 300 mM NaCl with or without laminaran. The intracellular fluorescence intensity of MitoSOX in Control (no treatment group) was 5654 ± 969 a.u., which was significantly lower than that of NaCl+ (NaCl treatment group) at 9016 ± 2035 a.u. The fluorescence intensity of NaCl + Lam (NaCl and laminaran treatment group) was 5843 ± 914 a.u., similar to that of Control (Figure 2a,b). The fluorescence intensities of HPF in Control and NaCl + Lam were 1048 ± 32 a.u. and 1250 ± 63 a.u., respectively. On the other hand, the fluorescence intensity in NaCl+ was 1760 ± 188 a.u. and this value was significantly higher than that of Control and NaCl + Lam. These results indicated that laminaran scavenges O_2_^−^, OH^−^, and ONOO^−^ were generated intracellularly by NaCl.

### 2.3. Laminaran Enhances Expression of Antioxidant Enzymes

**Figure 3:**
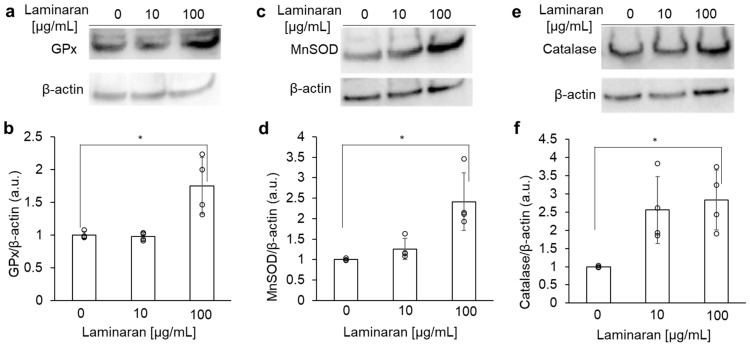
Antioxidant enzyme expression assay with laminaran using Western blot analysis. The cells were incubated in a medium containing 100 μg/mL laminaran for 24 h. Expression levels of GPx (**a**,**b**), MnSOD (**c**,**d**), and catalase (**e**,**f**) in RGM1 cells. β-actin was used as Control. Data are expressed as the mean ± SD (*n* = 4). * *p* < 0.05.

Western blotting was used to investigate the activation of antioxidant enzymes (such as glutathione peroxidase (GPx), manganese superoxide dismutase (MnSOD), and catalase) by laminaran in rat gastric epithelial cells. The activation of GPx, MnSOD, and catalase increased in a dose-dependent manner (Figure 3). Notably, activation of the three enzymes in 100 µg/mL laminaran-treated cells was significantly higher than the activation in laminaran-untreated cells.

### 2.4. Laminaran Suppresses NaCl-Derived Reduction in Mitochondrial Membrane Potential

**Figure 4:**
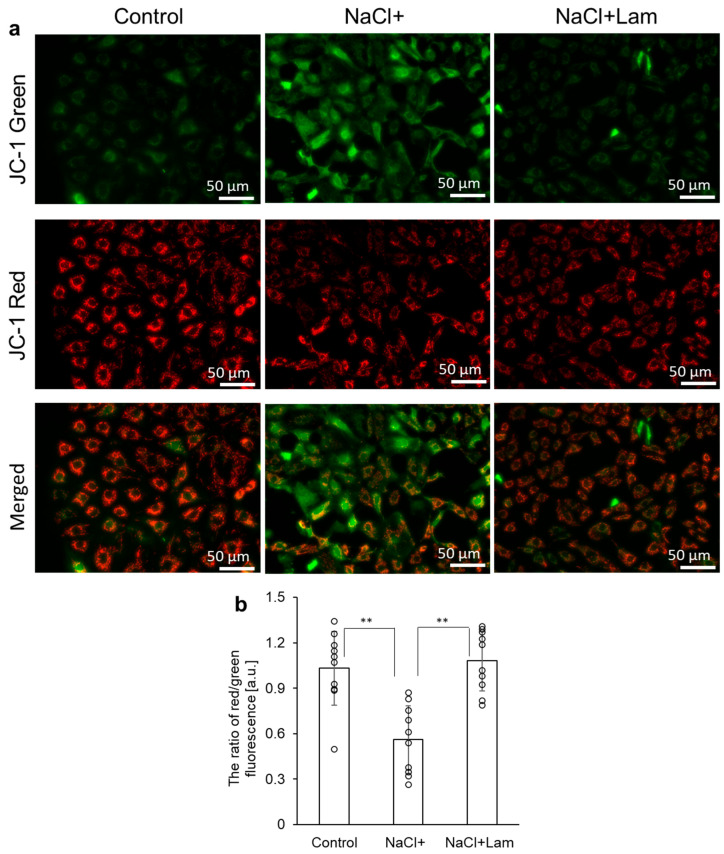
The fluorescence intensity of JC-1. The cells were incubated in a medium containing 300 mM NaCl with or without 100 µg/mL laminaran for 1 h. The fluorescence intensity of JC-1 was measured under the fluorescence microscopy. (**a**) Fluorescent microscopy utilized to assess cellular uptake of JC-1. (**b**) Data are expressed as means ± SD (*n* = 10). The sample size is the number of cells in the analyzed images. ** *p* < 0.01.

Mitochondrial membrane potential was observed under a fluorescence microscope using JC-1. The cells were incubated for 1 h in a medium containing 300 mM NaCl, with or without laminaran. The red/green fluorescence ratio of NaCl + Lam cells was similar to that of Control and higher than that of NaCl+ cells. These findings imply that NaCl treatment reduced mitochondrial membrane potential, whereas laminaran suppressed the NaCl-induced reduction in mitochondrial membrane potential (Figure 4).

### 2.5. Intracellular Fluorescence Intensity of Actin Filament

**Figure 5:**
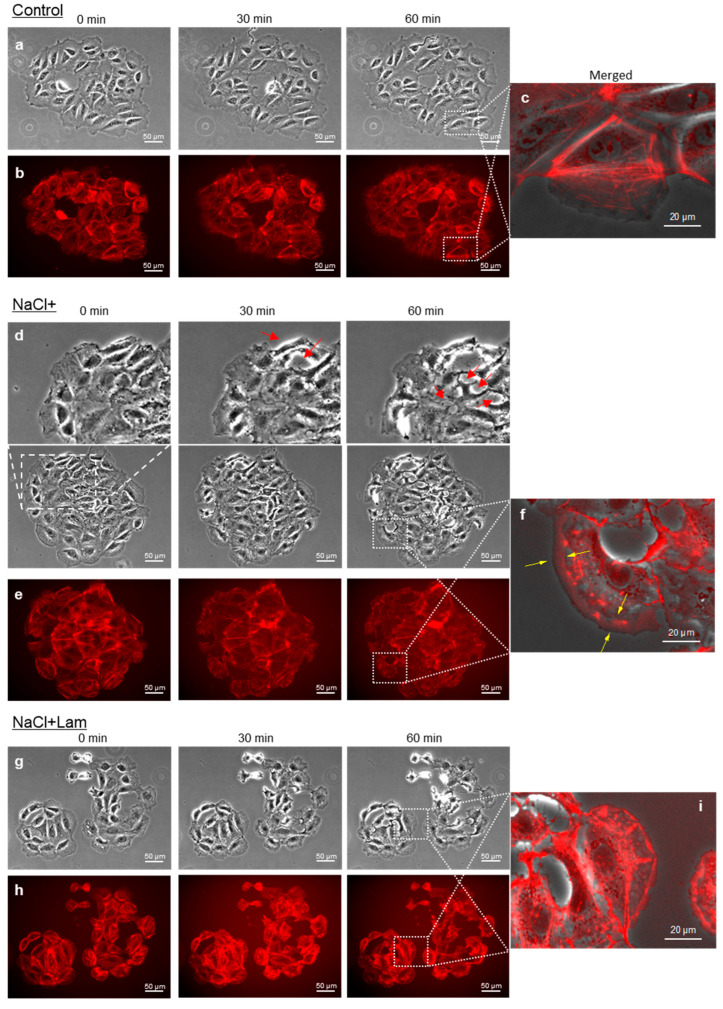
Visualization of cellular actin filaments. Phase contrast, fluorescence and merged images of Control cells shown in (**a**–**c**). Those of NaCl+ cells shown in (**d**–**f**). Those of NaCl + Lam cells shown in (**g**–**i**). Red arrow showed injured cells. Lamellipodia formation can be observed in NaCl+ (yellow arrow).

Actin filaments were visualized using SPY555-Act (Figure 5). Time-lapse imaging was performed 60 min after the treatment. No difference was observed between the phase contrast and fluorescence images in a time-dependent manner for Control. In Control cells, actin filaments were observed to bind myosin and maintain the cytoskeleton (Figure 5a,b) and were striated and located in the cell body, which was maintained after 60 min (Figure 5c). In NaCl+ cells, the actin filaments began to change in formation after the addition of NaCl, became band-shaped, and were located at the cell edge in almost all cells (Figure 5d,e). Cell shrinkage was observed from 30 min after NaCl treatment, with many cells detaching from the substrate after 60 min (Figure 5d,e). These morphological changes indicate that the cells were injured by NaCl. Lamellipodia, including a two-dimensional actin network, formed at the cell edges 30 min after NaCl treatment, and the area of lamellipodia at the cell edges increased after 60 min (Figure 5f). In NaCl + Lam cells, some actin filaments disassembled into small sizes 30 min after treatment, and, in some cells, the localization of actin filaments at the cell edges was observed after 60 min. However, these phenomena were less pronounced than those observed for NaCl+ cells (Figure 5g,h). Additionally, most of the actin filaments did not form peripheral bands, and lamellipodia formation was less common than in NaCl+ cells (Figure 5i).

### 2.6. Effect of NaCl on Cellular Elastic Modulus

**Figure 6:**
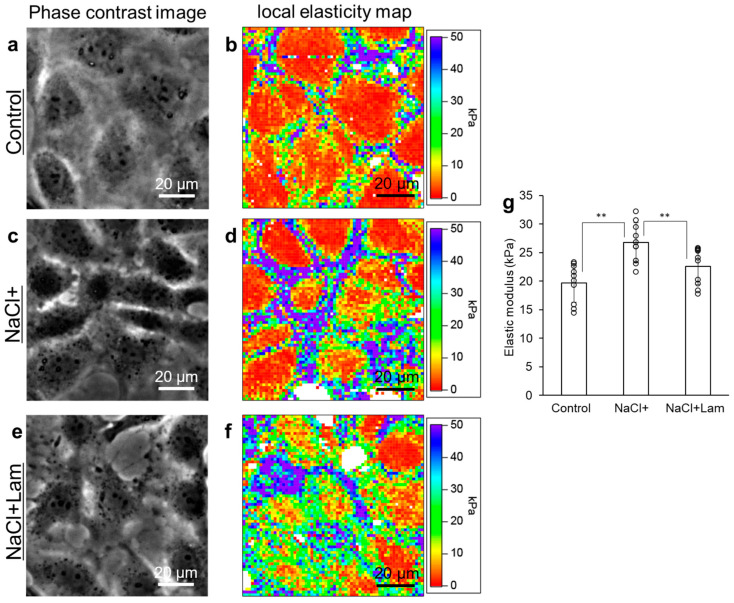
Measurement of cellular elastic modulus using AFM. The cells were incubated in a medium containing 300 mM NaCl with or without 100 μg/mL laminaran for 1 h. Phase contrast images (**a**,**c**,**e**) and local elasticity maps (**b**,**d**,**f**). (**a**,**b**): Control; (**c**,**d**): NaCl+; and (**e**,**f**): NaCl + lam. Average of the local elastic modulus at cell edges (**g**). Data are expressed as the mean ± SD (*n* = 10). ** *p* < 0.01.

Figure 6 shows the phase contrast images and local elasticity maps. Local elasticity maps are displayed in pseudo-colors, with red indicating a low elastic modulus, and purple indicating a high elastic modulus. No differences were observed in the phase contrast images of the three groups (Figure 6a,c,e). The pseudo-color of the cell edges in NaCl+ cells (Figure 6d) was more purple than the pseudo-color in Control (Figure 6b) and NaCl + Lam cells (Figure 6f). These results indicate that the local elastic modulus at the cell edge in NaCl+ cells was higher than in the Control and NaCl + Lam cells. In the local elasticity maps, the average local elastic modulus at the cell edge in NaCl+ cells was significantly higher than that in the Control and NaCl + Lam groups (Figure 6g).

## 3. Discussion

The effects of salt stress have been extensively investigated in both plant and animal cells. For terrestrial plants, salt is an environmental stressor of growth and development [19,20]. When plants sense salt stress, they respond to ionic and osmotic stresses and activate ROS signaling [19]. Salt stress, in turn, impairs many cellular functions such as cytoskeleton dynamics, ion balance, osmotic homeostasis, and ROS generation [21]. Moreover, salt stress induces ROS production in animal cells, particularly gastric cells, resulting in cytotoxicity [6]. We have previously investigated the relationship between gastric injury and ROS; specifically, we found that indomethacin and dabigatran induce cytotoxicity in gastric cells and tissue [22,23]. Cytotoxicity induced by these drugs is mediated by ROS and attenuated when combined with antioxidants [24,25]. Laminaran reduces cytotoxicity derived from indomethacin and dabigatran by scavenging ROS [18]. This study also revealed that similar to the reduction in drug-induced cytotoxicity, laminaran reduced NaCl-induced cytotoxicity (Figure 1) and scavenged NaCl-induced ROS, such as O_2_^−^, OH^−^, and ONOO^−^ (Figure 2), by activating antioxidant enzymes such as MnSOD, catalase, and GPx (Figure 3).

Mitochondria are the primary site of ROS generation in vivo. They possess their own DNA (mtDNA) encoding two rRNAs, 22 tRNAs, and 13 protein subunits, all of which are essential components of the oxidative phosphorylation system. Since mtDNA is more susceptible to damage caused by oxidative stress than nuclear DNA, a distinctive feature of the early stages of cell apoptosis is the disruption of mitochondria caused by changes in the mitochondrial membrane potential. Therefore, the mitochondrial membrane potential is a valuable indicator of cell health. Fluorescence microscopy with the membrane-permeant JC-1 dye is widely used in cell apoptosis studies to monitor mitochondrial health. The JC-1 dye facilitates discrimination between polarized and depolarized mitochondria; it exists as green fluorescent monomers at low membrane potentials but forms red fluorescent “J-aggregates” at higher potentials. Consequently, mitochondrial depolarization is indicated by a decrease in the red/green fluorescence intensity ratio [26]. In this study, the red/green fluorescence ratio in NaCl + Lam was significantly higher than that in NaCl+ (Figure 4). From this result, the following mechanism can be assumed: NaCl-induced ROS in the mitochondria first damaged mtDNA, lowered mitochondrial membrane potential, and changed mitochondrial oxidative phosphorylation, resulting in cell damage and death. However, simultaneous addition of laminaran suppressed intracellular ROS generation, leading to the sup-pression of NaCl-derived reduction in mitochondrial membrane potential, resulting in the attenuation of NaCl-derived cytotoxicity.

Intracellular ROS production induces the formation of stress fibers [27,28]. Since both lamellipodia and stress fibers are formed by actin filament [29], we used phase contrast and fluorescence microscopy to observe the effects of NaCl treatment on actin filament and whether this effect is alleviated by laminaran. As presented in Figure 5d–f, NaCl+ had significant effects on actin filaments, including actin filament reformation, localization to cell edges, and subsequent lamellipodia formation and expansion. Moreover, NaCl+ induced morphological changes by inducing cell contraction and suspension.

In contrast, the NaCl + Lam had effects on conditions of some actin filaments, such as degradation to a smaller size, localization at the cell edge, and lamellipodia formation (Figure 5g–i); however, many actin filaments were well retained, as observed in the Control. From these results, we concluded that lamellipodia was formed by NaCl+; however, lamellipodia formation was suppressed by the simultaneous addition of laminaran. Rac1 is involved in the formation of lamellipodia and RhoA induces the formation of actin filaments and polymerization to form stress fibers, and these proteins are antagonistic to each other [8]. As presented in Figure 5f, lamellipodia formation was observed upon NaCl+; however, no stress fiber formation was detected. Therefore, we hypothesize that NaCl treatment preferentially promotes Rac1 expression. Furthermore, although lamellipodia formation was observed in some cells treated with NaCl + Lam, the shape and position of the actin filaments were largely retained, as observed in the Control. The simultaneous addition of laminaran may have suppressed NaCl-derived Rac1 expression and promoted RhoA expression to levels close to those of the Control.

The reason for the activation of Rac1 by NaCl+ is assumed to be the involvement of Na/K-ATPase, which is a sodium transporter that utilizes ATP to maintain intracellular Na+ and K+ and is activated by an increase in NaCl concentration [30]. Na/K-ATPase interacts with proteins in the phosphatidylinositol 3-kinase (PI3-K) pathway, leading to cytoskeletal remodeling and lamellipodia formation by activating Rac1 [7,31,32]. As Rac1 can also stimulate the activation of PI3-K, NaCl exposure likely enhances this interaction and promotes the generation of lamellipodia and reorganization of the actin cytoskeleton. Additionally, we quantified the extent of lamellipodia formation using the AFM method that we previously developed [23] (as described in Section 4). As presented in Figure 6, the elastic modulus of NaCl+ at the cell edge was higher than that of the Control. The cellular elastic modulus is affected by the growth of actin filaments and stress fibers [27]. Therefore, the increase in the elastic modulus at the cell edge in NaCl+ suggests that a large number of actin filaments were localized at the cell edges, promoting lamellipodia formation. However, the pseudo-color at the cell edge in NaCl + Lam was comparable to that of the Control, and the elastic modulus of the cell edge in NaCl + Lam was almost the same, with little increase compared to the Control (Figure 6g). These results indicate that laminaran inhibits the NaCl-induced increase in cellular elastic modulus and the subsequent formation of lamellipodia.

Laminaran inhibits cell migration [33]. Owing to the promotion of lamellipodia formation providing an increase in migration ability [34], the migration-inhibiting effect of laminaran is thought to be due to the inhibition of lamellipodia formation caused by the inhibition of the downstream signal of Na/K-ATPase on cytoskeletal reconstruction. By suppressing the increase in intracellular Na+ concentration, laminaran may inhibit Na+ efflux via Na/K-ATPase, thereby inhibiting the formation of lamellipodia. Alternatively, laminaran may inhibit cytoskeletal reconstruction and lamellipodia formation, and suppress the PI3-kinase pathway, which is a downstream signal of Na/K-ATPase. Antioxidants also inhibit cell migration [35,36]. Resveratrol, an antioxidant, inhibits cell migration through PI3-K activation [37]. We have previously reported that laminaran scavenges ROS, and this study further demonstrated that laminaran scavenges NaCl-derived intracellular H_2_O_2_ by enhancing the expression of antioxidant enzymes (Figure 2 and Figure 3). Therefore, laminaran may inhibit cell migration by activating PI-3-K and inactivating NaCl-derived Na/K ATPase.

Finally, the structural characteristics of the laminaran used in this study warrant further consideration. The laminaran employed in our assays was derived from *Eisenia bicyclis*. While the primary backbone of laminaran consists of β-1,3/1,6-glucans, the specific ratio of 1,3- to 1,6-linkages in the current preparation was not fully elucidated. Determining the optimal structural ratio of laminaran required to effectively prevent NaCl-induced mucosal injury remains an important subject for future investigation.

## 4. Materials and Methods

### 4.1. Reagents

Laminaran (L0088), extracted from Eisenia bicyclis, was purchased from Tokyo Chemical Industry (Tokyo, Japan). NaCl (196-01671), Dulbecco’s Modified Eagle Medium (DMEM)/F12 (042-30555), and penicillin/streptomycin (168-23191) were from Wako Pure Chemical Industries (Osaka, Japan). Fetal bovine serum (Hyclone) was from GE Healthcare Science (Marlborough, MA, USA), Cell Counting Kit-8 (CCK8) (CK04) was from Dojindo (Tokyo, Japan), and 2-[6-(4′-hydroxy)phenoxy-3H-xanthen-3-on-9-yl] benzoic acid (HPF) (SK3001-01) was from Goryo Chemical (Sapporo, Japan). FluoroBrite DMEM (A18967-01), MitoSOX (M36008), NuPAGE LDS Sample Buffer (NP0008) were from Thermo Fisher Scientific (Waltham, MA, USA). BCA protein assay kit (T9300A) was purchased from Takara Bio Inc. (Shiga, Japan). EzWestBlue W (WSE-7110) was purchased from Atto Corporation (Tokyo, Japan). Polyacrylamide gel (SDG-545) was from BIO CRAFT Co. Ltd. (Tokyo, Japan), and polyvinylidene difluoride (PVDF) (1620177) membrane was from Bio-Rad Laboratories (Hercules, CA, USA). Can Get Signal^®^ PVDF blocking reagent (NYPBR01), Can Get Signal^®^ immunoreaction enhancer solution 1 (NKB-201), and Can Get Signal^®^ immunoreaction enhancer solution 2 (NKB-301) were from TOYOBO CO. Ltd. (Osaka, Japan). Primary antibody of manganese superoxide dismutase (MnSOD) (13141), catalase (14097), β-actin (4967), and secondary horseradish peroxidase (HRP)-linked anti-rabbit IgG antibody (7074) were from Cell Signaling Technology (Tokyo, Japan). GPx (ab22604) was from Abcam (Tokyo, Japan). SPY555-Actin (CY-SC202) was from Cytoskeleton Inc. (Denver, CO, USA).

### 4.2. Cell Culture

The rat gastric epithelial cell line RGM1 was purchased from the Riken Cell Bank (Tsukuba, Japan). RGM1 cells were cultured in DMEM/F12 with L-glutamine. The culture medium contained 10% heat-inactivated fetal bovine serum and 1% penicillin/streptomycin. Cells were cultured in a CO_2_ incubator containing 5% CO_2_ at 37 °C. All experiments were repeated at least three times.

### 4.3. Cell Viability Assay

Cell viability was assessed using the CCK8. Cells were cultured in 96-well plates at a density of 5 × 10^3^ cells/well and incubated for two days. The supernatant was aspirated, and the cells were cultured in a medium containing 280 or 300 mM NaCl for 24 h. After cultivation, the cells were incubated with 10% CCK8. Absorbance was measured at 450 nm using a Synergy H1 microplate reader (BioTek Instruments Inc., Winooski, VT, USA). The efficacy of laminaran against NaCl-induced cytotoxicity was evaluated using CCK8 as follows: cells were cultured in 96-well plates at a density of 5 × 10^3^ cells/well and incubated for two days. The supernatant was aspirated, and cells were cultured for 24 h in medium containing 0, 10, and 100 µg/mL laminaran respectively, with 300 mM NaCl. After cultivation, the cells were incubated with 10% CCK8.

### 4.4. Measurement of Intracellular O_2_^−^ Production

MitoSOX was used as a fluorescence dye to detect the intracellular O_2_^−^ production [38]. RGM1 cells were cultured in 24-well plates at a density of 1 × 10^4^ cells/well and incubated for two days. After aspiration of the supernatant, RGM1 cells were incubated for 1 h at 37 °C in medium containing 0 or 100 µg/mL laminaran with 300 mM NaCl. The groups without NaCl, with NaCl, and with NaCl + Laminaran were Control, NaCl+, and NaCl + Lam, respectively. The cells were then incubated with 5 μM MitoSOX in FluoroBrite DMEM for 10 min. The fluorescence intensity of cells was measured using a fluorescence microscope (IX83; Olympus, Tokyo, Japan) with a PlanAPO 20× objective lens (Olympus). MitoSOX was excited using a 535–555 nm filter, and the emission was measured using a 570–625 nm filter. Fluorescence intensities were analyzed using ImageJ version 1.54k, an open-access software developed by the National Institute of Health. The region of interest of the cells in the image was selected and the fluorescence intensities of each cell were analyzed.

### 4.5. Measurementn of Intracellular OH^−^ and ONOO^−^ Production

Intracellular OH^−^ and ONOO^−^ production was detected using the fluorescent dye HPF [39]. RGM1 cells were cultured in 24-well plates at a density of 1 × 10^4^ cells/well and incubated for two days. After aspiration of the supernatant, RGM1 cells were incubated for 1 h at 37 °C in a medium containing 0 or 100 µg/mL laminaran, in addition to 300 mM NaCl. The cells from the supernatant were incubated with 5 μM HPF in Fluobright for 15 min to incorporate HPF into the cells, following which the medium was replaced with Fluobright. The fluorescence intensity of cells in each treatment group was measured using a fluorescence microscope (IX83) with a PlanAPO 20× objective lens. The HPF was excited using a 460–495 nm filter, and the emission was measured using a 510–550 nm filter. Fluorescence intensities were analyzed using ImageJ. The region of interest of the cells in the image was selected and the fluorescence intensities of each cell were analyzed.

### 4.6. Western Blot Assay

RGM1 cells were cultured for two days in DEME/F12 medium in 60 mm dishes. The cells were then exposed to 0, 1, and 100 µg/mL laminaran for 24 h. The cells were washed three times with PBS, followed by the addition of RIPA buffer containing 25 mM Tris-HCl (pH 7.6), 150 mM NaCl, 1% (*v*/*v*) Triton X-100, 0.1% (*w*/*v*) SDS, and 0.2% (*w*/*v*) deoxycholic acid to the cells placed on ice to facilitate cell lysis. The whole cell lysates obtained were used for Western blotting analysis. The amount of total protein was standardized using BCA protein assay kit. Samples were diluted in NuPAGE LDS Sample Buffer and boiled in a water bath for 10 min at 70 °C. The samples were added to the wells for sodium dodecyl sulfate–polyacrylamide gel electrophoresis. The 15% gels were electrophoresed at 100 V for 60 min, and the proteins were transferred to a PVDF membrane by electrophoresis at 1.2 mA/cm^2^ for 60 min. The membrane was blocked for 60 min using PVDF blocking reagent from Can Get Signal^®^ and probed with primary and secondary antibodies. Anti-rabbit antibodies (1:1000) against GPx, MnSOD, and catalase were added to Can Get Signal^®^ immunoreaction enhancer solution 1, and the membranes were exposed overnight. The primary antibody solution was aspirated, and the membrane was washed three times with PBS containing Tween 20. The secondary HRP-linked anti-rabbit IgG antibody (1:1000) was added to Can Get Signal^®^ immunoreaction enhancer solution 2, and the membrane was exposed for 60 min. The membranes were visualized using Lumina Forte Western HRP substrate (Millipore Co., Billerica, MA, USA). Images of the blots were captured using Fusion FX7 Edge imaging system (Vilber Lourmat, Co., Marne-la-Vallée, France). β-actin was used as the Control for protein concentration estimation.

### 4.7. Evaluation of Mitochondrial Membrane Potential Using JC-1

The change in mitochondrial membrane potential induced by NaCl in the presence and absence of laminaran was measured using JC-1 (5,5′,6,6′-tetrachloro-1,1′,3,3′-tetraethyl benzimidazolyl carbocyanine iodide/chloride) [40]. Cells were cultured in 24-well plates at 1 × 10^4^ cells/well and incubated for two days. The supernatant was aspirated, and the cells were incubated for 1 h in 300 mM NaCl containing medium with or without 100 µg/mL laminaran. After incubation, the supernatant was aspirated, and the cells were incubated for 30 min in a medium containing 2 μM JC-1. The supernatant was aspirated, and the cells were rinsed twice with PBS, and FluoroBrite DMEM was added. The fluorescence intensity of cells in each treatment group was measured using a fluorescence microscope (IX83) with a PlanAPO 20× objective lens. JC-1 red fluorescence excitation was induced at 535–555 nm, and the emission spectra were recorded at 570–625 nm. JC-1 green fluorescence excitation was induced at 460–480 nm, and the emission spectra were recorded at 495–540 nm. In living cells, JC-1 is taken up by the mitochondria and emits red fluorescence. However, in apoptotic cells, it is not taken up by the mitochondria and emits green fluorescence in the cytoplasm. Therefore, the red/green ratio was measured to evaluate mitochondrial membrane potential. Fluorescence intensities were analyzed using ImageJ. The region of interest of the cells in the image was selected and the fluorescence intensities of each cell were analyzed.

### 4.8. Fluorescence Observation of Cellular Actin Filament

SPY555-Actin (Cytoskeleton, Inc.) was used as the fluorescent dye to detect cellular actin filaments. RGM1 cells were cultured in a 35 mm glass-bottom dish and incubated for 2 days. The supernatant was aspirated, and the RGM1 cells were incubated in a medium containing SPY555-Actin (1:1000 dilution) for 1 h. The supernatant was aspirated, and the cells were incubated in medium containing 300 mM NaCl and SPY555-Actin (1:2000 dilution) with or without 100 µg/mL laminaran. Time-lapse observations were performed using a desktop incubator (Tokai Hit, Inc. Shizuoka, Japan)) equipped with a fluorescence microscope (IX83) with a PlanAPO 20× objective lens. SPY555-Actin was excited using a 535–555 nm filter, and the emission was measured using a 570–625 nm filter.

### 4.9. Measurement of Cellular Elastic Modulus Using Atomic Force Microscopy (AFM)

We conducted AFM following a previously demonstrated method [27]. RGM1 cells were cultured in 60 mm dishes and incubated for 2 days. After aspiration of the supernatant, RGM1 cells were incubated for 1 h in medium containing 300 mM NaCl with or without 100 µg/mL laminaran. After incubation, the medium was replaced with fresh medium. The elastic modulus of the cells was measured using the atomic force microscope observations made using an Asylum Research MFP-3D-BIO AFM (Oxford Instruments, Santa Barbara, CA, USA) mounted on an IX71 system (Olympus). The AFM measurements were performed using an Olympus Biolever BL-AC40TS-C2 cantilever (silicon nitride, spring constant of 0.1 N/m, half-apex angle of 17.5°). The force curve was measured on a grid of 64 × 64 points in an area of 100 × 100 μm, and the local elastic modulus was determined from the force curve at each point using Hertz’s equation [41,42]. This was used as an elastic modulus map. The elastic modulus of the cantilever was 290 GPa, while the Poisson ratio of the sample was 0.5, calculated using Hertz’s equation. Approximately five to seven cells were observed in an area of 100 × 100 μm, and the average elastic modulus of each cell was calculated by dividing the cell into two regions: the region near the nucleus and the rest of the cell (cell body). The region with an elastic modulus of 150 kPa or more was defined as the dish portion and was excluded from the average elastic modulus of the cell body.

### 4.10. Statistical Analysis

Data were expressed as the mean ± standard deviation and assessed using analysis of variance. Individual groups were compared using Tukey’s post hoc test with *p* < 0.05 considered significant.

## 5. Conclusions

NaCl induced cytotoxicity in rat gastric epithelial cell line RGM1 and promoted intracellular ROS production. Laminaran, an antioxidant, can attenuate cytotoxicity by reducing intracellular ROS production derived from excess NaCl intake and suppressing the NaCl-derived reduction in mitochondrial membrane potential. NaCl promoted lamellipodia formation, which was inhibited when NaCl was combined with laminaran. The ingestion of laminaran-rich seaweeds, such as kelp, may improve health disorders caused by high-salt diets.

## Data Availability

The original contributions presented in this study are included in the article.

## References

[1] GBD 2017 Diet Collaborators (2019). Health effects of dietary risks in 195 countries, 1990–2017: A systematic analysis for the global burden of disease study 2017. Lancet.

[2] Rust P., Ekmekcioglu C. (2017). Impact of salt intake on the pathogenesis and treatment of hypertension. Adv. Exp. Med. Biol..

[3] Tsugane S. (2005). Salt, salted food intake, and risk of gastric cancer: Epidemiologic evidence. Cancer Sci..

[4] Tsugane S., Sasazuki S., Kobayashi M., Sasaki S. (2004). Salt and salted food intake and subsequent risk of gastric cancer among middle-aged japanese men and women. Br. J. Cancer.

[5] Bray F., Ferlay J., Soerjomataram I., Siegel R.L., Torre L.A., Jemal A. (2018). Global cancer statistics 2018: Globocan estimates of incidence and mortality worldwide for 36 cancers in 185 countries. CA A Cancer J. Clin..

[6] Tamura M., Matsui H., Nagano Y.N., Kaneko T., Indo H.P., Majima H.J., Hyodo I. (2013). Salt is an oxidative stressor for gastric epithelial cells. J. Physiol. Pharmacol..

[7] Kaplan J.H. (2005). A moving new role for the sodium pump in epithelial cells and carcinomas. Sci. STKE.

[8] Rottner K., Stradal T.E. (2011). Actin dynamics and turnover in cell motility. Curr. Opin. Cell Biol..

[9] Friedl P., Gilmour D. (2009). Collective cell migration in morphogenesis, regeneration and cancer. Nat. Rev. Mol. Cell Biol..

[10] Tokuraku K., Kuragano M., Uyeda T.Q.P. (2020). Long-range and directional allostery of actin filaments plays important roles in various cellular activities. Int. J. Mol. Sci..

[11] Rotsch C., Radmacher M. (2000). Drug-induced changes of cytoskeletal structure and mechanics in fibroblasts: An atomic force microscopy study. Biophys. J..

[12] Blanchoin L., Boujemaa-Paterski R., Sykes C., Plastino J. (2014). Actin dynamics, architecture, and mechanics in cell motility. Physiol. Rev..

[13] Shannon E., Abu-Ghannam N. (2019). Seaweeds as nutraceuticals for health and nutrition. Phycologia.

[14] Usoltseva R.V., Belik A.A., Kusaykin M.I., Malyarenko O.S., Zvyagintseva T.N., Ermakova S.P. (2020). Laminarans and 1,3-β-D-glucanases. Int. J. Biol. Macromol..

[15] Wouk J., Dekker R.F.H., Queiroz E.A.I.F., Barbosa-Dekker A.M. (2021). Β-glucans as a panacea for a healthy heart? Their roles in preventing and treating cardiovascular diseases. Int. J. Biol. Macromol..

[16] Suzuki T., Kusano K., Kondo N., Nishikawa K., Kuge T., Ohno N. (2021). Biological activity of high-purity β-1,3-1,6-glucan derived from the black yeast aureobasidium pullulans: A literature review. Nutrients.

[17] Kadam S.U., Tiwari B.K., O’Donnell C.P. (2015). Extraction, structure and biofunctional activities of laminarin from brown algae. Int. J. Food Sci. Technol..

[18] Kurokawa H., Marella T.K., Matsui H., Kuroki Y., Watanabe M.M. (2023). Therapeutic potential of seaweed-derived laminaran: Attenuation of clinical drug cytotoxicity and reactive oxygen species scavenging. Antioxidants.

[19] Zhao S., Zhang Q., Liu M., Zhou H., Ma C., Wang P. (2021). Regulation of plant responses to salt stress. Int. J. Mol. Sci..

[20] Yu Z., Duan X., Luo L., Dai S., Ding Z., Xia G. (2020). How plant hormones mediate salt stress responses. Trends Plant Sci..

[21] Park H.J., Kim W.Y., Yun D.J. (2016). A new insight of salt stress signaling in plant. Mol. Cells.

[22] Yoshikawa T., Naito Y., Kishi A., Tomii T., Kaneko T., Iinuma S., Ichikawa H., Yasuda M., Takahashi S., Kondo M. (1993). Role of active oxygen, lipid peroxidation, and antioxidants in the pathogenesis of gastric mucosal injury induced by indomethacin in rats. Gut.

[23] Charlton B., Redberg R. (2014). The trouble with dabigatran. BMJ.

[24] Saito R., Tamura M., Matsui H., Nagano Y., Suzuki H., Kaneko T., Mizokami Y., Hyodo I. (2015). Qing dai attenuates nonsteroidal anti-inflammatory drug-induced mitochondrial reactive oxygen species in gastrointestinal epithelial cells. J. Clin. Biochem. Nutr..

[25] Kurokawa H., Taninaka A., Shigekawa H., Matsui H. (2021). Dabigatran etexilate induces cytotoxicity in rat gastric epithelial cell line via mitochondrial reactive oxygen species production. Cells.

[26] Kumar B., Koul S., Khandrika L., Meacham R.B., Koul H.K. (2008). Oxidative stress is inherent in prostate cancer cells and is required for aggressive phenotype. Cancer Res..

[27] Taninaka A., Ugajin S., Kurokawa H., Nagoshi Y., Kamiyanagi M., Takeuchi O., Matsui H., Shigekawa H. (2022). Direct analysis of the actin-filament formation effect in photodynamic therapy. RSC Adv..

[28] Taninaka A., Kurokawa H., Kamiyanagi M., Ochiai T., Arashida Y., Takeuchi O., Matsui H., Shigekawa H. (2023). Polphylipoprotein-induced autophagy mechanism with high performance in photodynamic therapy. Commun. Biol..

[29] Ridley A.J., Schwartz M.A., Burridge K., Firtel R.A., Ginsberg M.H., Borisy G., Parsons J.T., Horwitz A.R. (2003). Cell migration: Integrating signals from front to back. Science.

[30] Liu J., Xie Z.J. (2010). The sodium pump and cardiotonic steroids-induced signal transduction protein kinases and calcium-signaling microdomain in regulation of transporter trafficking. Biochim. Biophys. Acta.

[31] Barwe S.P., Anilkumar G., Moon S.Y., Zheng Y., Whitelegge J.P., Rajasekaran S.A., Rajasekaran A.K. (2005). Novel role for na,k-atpase in phosphatidylinositol 3-kinase signaling and suppression of cell motility. Mol. Biol. Cell.

[32] Welch H.C., Coadwell W.J., Stephens L.R., Hawkins P.T. (2003). Phosphoinositide 3-kinase-dependent activation of rac. FEBS Lett..

[33] Desamero M.J., Kakuta S., Chambers J.K., Uchida K., Hachimura S., Takamoto M., Nakayama J., Nakayama H., Kyuwa S. (2018). Orally administered brown seaweed-derived β-glucan effectively restrained development of gastric dysplasia in a4gnt ko mice that spontaneously develop gastric adenocarcinoma. Int. Immunopharmacol..

[34] Yan Y., Tsukamoto O., Nakano A., Kato H., Kioka H., Ito N., Higo S., Yamazaki S., Shintani Y., Matsuoka K. (2015). Augmented ampk activity inhibits cell migration by phosphorylating the novel substrate pdlim5. Nat. Commun..

[35] McCall B., McPartland C.K., Moore R., Frank-Kamenetskii A., Booth B.W. (2018). Effects of astaxanthin on the proliferation and migration of breast cancer cells in vitro. Antioxidants.

[36] Jang S.Y., Kim J., Hong E., Lee K., Na Y., Yeom C.H., Park S. (2023). Curcumin inhibits human cancer cell growth and migration through downregulation of svct2. Cell Biochem. Funct..

[37] Sun Y., Zhou Q.M., Lu Y.Y., Zhang H., Chen Q.L., Zhao M., Su S.B. (2019). Resveratrol inhibits the migration and metastasis of mda-mb-231 human breast cancer by reversing tgf-β1-induced epithelial-mesenchymal transition. Molecules.

[38] Robinson K.M., Janes M.S., Pehar M., Monette J.S., Ross M.F., Hagen T.M., Murphy M.P., Beckman J.S. (2006). Selective fluorescent imaging of superoxide in vivo using ethidium-based probes. Proc. Natl. Acad. Sci. USA.

[39] Setsukinai K., Urano Y., Kakinuma K., Majima H.J., Nagano T. (2003). Development of novel fluorescence probes that can reliably detect reactive oxygen species and distinguish specific species. J. Biol. Chem..

[40] Salvioli S., Ardizzoni A., Franceschi C., Cossarizza A. (1997). Jc-1, but not dioc6(3) or rhodamine 123, is a reliable fluorescent probe to assess Δψ changes in intact cells: Implications for studies on mitochondrial functionality during apoptosis. FEBS Lett..

[41] Sneddon I.N. (1965). The relation between load and penetration in the axisymmetric boussinesq problem for a punch of arbitrary profile. Int. J. Eng. Sci..

[42] Rosenbluth M.J., Lam W.A., Fletcher D.A. (2006). Force microscopy of nonadherent cells: A comparison of leukemia cell deformability. Biophys. J..

